# What Makes a National Pharmaceutical Track and Trace System Succeed? Lessons From Turkey

**DOI:** 10.9745/GHSP-D-20-00084

**Published:** 2020-09-30

**Authors:** Koray Parmaksiz, Elizabeth Pisani, Maarten Olivier Kok

**Affiliations:** a Erasmus School of Health Policy & Management, Erasmus University Rotterdam, Rotterdam, Netherlands.; b Policy Institute, King’s College London, London, United Kingdom.; c Department of Health Sciences, Vrije Universiteit Amsterdam, Amsterdam, Netherlands

## Abstract

**Background:**

Track and trace systems are increasingly being implemented as a technological solution to secure pharmaceutical supply chains. Turkey was the first country to implement a full pharmaceutical track and trace system throughout the entire regulated domestic supply chain. This article explores the emergence and functioning of this system and the consequences for substandard and falsified medicine with a focus on the underlying political and economic factors.

**Methods:**

This study uses an explanatory case study approach that combined interviews with purposefully selected key informants and document analyses.

**Results:**

The main drivers for implementing the pharmaceutical track and trace system in Turkey centered on the elimination of reimbursement fraud and the prevention of falsified medicine in the regulated supply chain. Although stakeholders experienced both physical and software-related problems in implementation, the alignment of incentives of all stakeholders with the power of the state, along with leeway for adaptations, ultimately resulted in a successful process. This track and trace system provides a clean regulated supply chain, minimizes reimbursement fraud, facilitates fast market recalls, and can flag likely medicine shortages. Staff previously engaged in pharmacy inspections now concentrate on ensuring production quality, which reduces the risk of substandard medicines.

**Conclusions:**

In Turkey, 4 factors drove the successful implementation of pharmaceutical track and trace: the political determination to eliminate reimbursement fraud, a large pharmaceutical market dominated by a single payer, medicine reimbursement being contingent on verified dispensing and prescription, and flexibility to adapt the system according to the needs of stakeholders during implementation.

## Abstract (precis)

Successful implementation of a pharmaceutical track and trace system depended on the political determination to eliminate reimbursement fraud, as well as establishing a pharmaceutical market dominated by a single payer, making reimbursement contingent on verified dispensing and prescription, and being flexible in adapting the system according to stakeholders’ needs.

## Teaser

**Key Findings:**

- Turkey implemented the world's first full pharmaceutical track and trace system.
- Its success depended on 4 factors:
  - Political determination
  - Industry incentives
  - Reimbursement linked to verified dispensing
  - Flexible implementation

**Key Implications:**

- Policy makers should perform a systemic analysis of market, political, economic, technical, and legal factors before implementing any pharmaceutical track and trace system.
- To achieve the intended outcomes, system design must align with the goals of implementation (e.g., to tackle fraud, reduce falsified medicine, minimize shortages). The system must be feasible in the local political and economic context.

## INTRODUCTION

Track and trace systems are logistical technologies that enable localizing and following a product throughout a supply chain^1^; they are used in many sectors, including aviation and retailing.^1^^,^^2^ In 2012, Turkey became the first country in the world to implement a full track and trace system to secure its domestic pharmaceutical supply chain. A growing number of countries are now following suit. Argentina and Saudi Arabia are among the countries that have already put such a system in place, while other countries, including China, the United States, and European Union (EU) member states, are currently in the process of implementation.^3^^–^^5^

For pharmaceuticals, 2 track and trace systems dominate. The first, known as a “point-of-dispense check” system, validates medicine packages at the points where they are dispensed to patients (e.g., pharmacy or hospital) with the code assigned during the manufacturing process. Other transactions (e.g., between wholesalers and distributors) are not systematically recorded.^3^ The European Medicines Verification System, implemented by the EU in February 2019 as part of the Falsified Medicines Directive, provides an example.^4^ This system allows for verifying the authenticity of the product, but not for tracking the product throughout the supply chain.^1^^,^^4^ The second track and trace system, often referred to as full track and trace, validates a medicine package at every stage of its journey through the supply chain. This system is in use in Turkey.^4^ Although the full track and trace system is more complex to implement, it provides additional potential benefits to those of the point-of-dispense check system. These benefits include real-time tracking throughout the entire supply chain, stock management for timely detection and prevention of stock-outs, targeted product recalls, and reduction of reimbursement fraud, theft, and medication errors.^1^^,^^6^^–^^9^

Recent studies show that the opportunity to enter the pharmaceutical supply chain differs between substandard and falsified medicines. Sub-standard medicines, which are defined as authorized products that fail to meet quality standards, enter the supply chain through manufacturers who might sacrifice quality to maximize profits.^10^^,^^11^ Falsified medicines, which have a deliberately misrepresented identity, composition, or source, are often introduced by criminals who see a market opportunity when shortages of quality and affordable medicine occur in the regulated market.^9^^,^^11^

Turkey began with a point-of-dispense check system known as *Ilaç Takip Sistemi* (ITS) in 2010, before introducing full track and trace in 2012.^3^^,^^8^ From the start, all medicines sold in Turkey had to be equipped with a DataMatrix code, which is a 2-dimensional barcode. A DataMatrix code contains information on the Global Trade Item Number, a serial number, an expiration date, and a batch number, which enables tracking the history and location of each medicine through the supply chain.^12^^,^^13^

### Implementing New Technologies

In this case study, we draw upon insights into the implementation of new technologies, which are based upon the rich literature on technological innovation. First, technological changes do not take place in a vacuum; they are embedded in dynamic and complex systems and are shaped by social, political, and economic factors. Therefore, when analyzing the implementation and functioning of new technologies, one must consider the context in which they are embedded.^14^^–^^16^

Technological changes are embedded in dynamic and complex systems and are shaped by social, political, and economic factors.

Second, initiation or successful implementation of technological change is often contingent on the determination of key actors to solve a perceived problem. The process of problematization and the emergence of a relative consensus about the nature of a problem are important first steps in aligning the incentives of all parties playing a key role in implementing the new technology.^17^

Third, implementation and innovation are intimately and reciprocally connected, which means that innovations transform during implementation.^18^ As a result, the technology that ultimately gets implemented often deviates from the initial plan. These adaptations, which are often omitted in retrospective accounts about the success of a technology, enable a certain system or technology to operate successfully within its specific context. Overlooking these adaptations undermines the successful reproducibility of technological innovations. In addition, adaptations enable different actors to assign different roles to the technology that are not limited to the official function of the technology.^19^ This results in unforeseen outcomes of the technology that are valuable to capture in order to reach its full potential.

Although Turkey was the first country to implement a full track and trace system, neither how the country achieved this significant feat nor what made it possible has been closely investigated. The aim of this study was to gain insight into political and economic factors that drove the implementation of the pharmaceutical track and trace system in Turkey. We paid special attention to the consequences of the system for substandard and falsified medicines. Insights from our study may provide valuable knowledge to other countries aiming to implement pharmaceutical track and trace systems and may contribute to the understanding of implementing large technological systems in the health sector.

## METHODS

### Study Design

For this qualitative case study, document analysis and semistructured interviews were carried out. We used an explanatory case study approach, the main purpose of which was “to explain how and why some conditions came to be.”^20^ Such an approach allows for investigating underlying factors that are often too complex to be captured by surveys or other quantitative measures alone.^21^ We believe that a detailed understanding of the implementation and adaptation of the pharmaceutical track and trace system will provide valuable in-sights into the complexities involved in implementing such large-scale health technologies.

Understanding of the implementation and adaptation of Turkey’s pharmaceutical track and trace system will elucidate the complexities of implementing such large-scale health technologies.

### Study Participants

The study participants were 16 purposefully selected key informants. Selection was based on their knowledge and expertise in political and economic factors influencing the emergence, implementation, and functioning of ITS. The aim of purposeful sampling is to increase depth and richness of the collected data by identifying and selecting information-rich cases from different perspectives.^22^^,^^23^ We sought to involve stakeholders across the supply chain, together with independent experts, to achieve a comprehensive evaluation of the pharmaceutical track and trace system in Turkey. Backgrounds of study participants are shown in the Table.

**TABLE.** Participants in the Qualitative Case Study of the Pharmaceutical Track and Trace System in Turkey, N=16

**Participant Category**	**No.**
Pharmaceutical manufacturers	2
Wholesalers	1
Health care providers	1
Ministry of Health/regulators	3
Technical agencies	
International organizations	1
Software developers	2
Associations/unions	3
Reimbursement agencies	2
Academics	1

### Data Collection Methods

To prepare for interviews and to triangulate findings, we reviewed relevant policy documents that helped us understand the emergence, implementation, and functioning of ITS, such as regulations on recall,^24^ labelling, package leaflets, tracing of human medicinal products,^13^^,^^25^ and ITS guidelines.^26^

Semistructured interviews were carried out between March and April 2018, using an interview guide (Supplement 1). We obtained written informed consent for interviews and requested permission to audio-record them. Three respondents refused to be audio-recorded, so detailed notes were instead taken during these interviews. Participants were anonymized using identification numbers that were stored separately from the study data. Interviews were conducted both in English and Turkish and lasted between 60 and 120 minutes.

### Data Analysis Methods

A constant comparative method of analysis was carried out. This method involves an iterative process, in which newly collected data in the form of interviews were triangulated with existing data from previous interviews, studies, or reports ob-tained during literature research to inform subsequent data collection and verify findings.^27^ First, the semistructured interviews were recorded and transcribed verbatim. Then, if necessary, they were translated into English. Interviews were coded using a coding structure jointly developed by the research team. This structure was based on political and economic factors enabling market opportunities for substandard and falsified medicine, along with factors facilitating or obstructing the implementation and functioning of track and trace systems (Supplement 2). Emerging themes and the analysis were discussed during 6 weekly team meetings, until consensus was reached. NVivo (12.0.0) was used as the qualitative data analysis software.

## RESULTS

### Historical Developments and Pricing Policies

To provide the contextual background, we asked participants to reflect on the historical developments of the health sector and the pharmaceutical industry in Turkey. Until the early 2000s, Turkey experienced several problems in the health sector, including insufficient insurance coverage, poor health outcomes such as life expectancy and maternal mortality, and relatively low governmental health expenditure.

We asked participants to reflect on the historical developments of the health sector and the pharmaceutical industry in Turkey.

In addition, Turkey had 3 state institutions, which are known by their Turkish abbreviations SSK, BAĞ-KUR, and Emekli Sandığı, providing health insurance to different employment-based groups. These institutions operated independently, which resulted in high fragmentation of service provision and restricted access to health services^28^^,^^29^:

*At that time about half of Turkey’s population, 50 percent was covered under SSK, and the number of hospitals those people could use was only 120. For the whole of Turkey, can you imagine? Half of the population in Turkey is doomed to only 120 hospitals. —*Academic

After the national elections in 2002, the Jus-tice and Development Party came into power in Turkey. This was the first time a political party with religious roots came into power as a single-party government since the establishment of the constitutionally secular Republic of Turkey. Therefore, they had to establish political legitimacy among their citizens and the international community. The government made a strong commitment to universal health coverage as a way of establishing political legitimacy among the country’s citizens and in the international community. The government subsequently introduced the Health Transformation Program in 2003 with the aim to increase insurance coverage and financial risk protection.

After 2006, the government merged the 3 state institutions providing health insurance to form a single-payer institution, called *Sosyal Güvenlik Kurumu* (SGK).^30^ Insurance coverage provided by SGK increased access to health services in Turkey considerably, resulting in a significant increase in public health expenditure. In response, the government introduced price-cutting measures, such as reference pricing in 2004 and global budgeting between 2010 and 2012. Despite these measures, manufacturers continued to supply the Turkish market, mainly because increased access to health services increased the overall volume of sales, creating a substantial pharmaceutical market that manufacturers were not willing to give up:

*There was not such a thing as convincing. The state is not obliged to convince. The customer is king. “I pay the money; I determine the conditions.” Turkey has such an advantage. I buy more than 80 percent of the market. They say: “If you are willing to give [medicines] under these conditions, then you can give them. Otherwise, I’m sorry, go sell them in another country, don’t sell them to me.” —*Multinational manufacturer

After 2006, the government merged the 3 state institutions providing health insurance to form the single-payer institution *Sosyal Güvenlik Kurumu* (SGK).

Respondents were asked if downward price pressures incentivized manufacturers to cut corners, resulting in substandard medicines. Both manufacturers and the Ministry of Health (MOH) emphasized that the production or import of substandard medicine in the Turkish market was very unlikely because Turkey has well-defined legislation and regulations, including Good Manufact-uring Practices (GMP), inspections, laboratories, and a pharmaceutical track and trace system. According to respondents, this strong regulatory framework minimized the possibility of substandard products on the market, while enabling rapid detection.

### Pharmaceutical Track and Trace

An MOH official explained that before the introduction of the pharmaceutical track and trace system in 2010, quality assurance of medical products was mainly based on market surveillance and inspections. When pharmacists, health professionals, or others reported suspicions about a product, the MOH might sample that product for testing. This largely reactive system was time and resource intensive.

Before the introduction of the pharmaceutical track and trace system, quality assurance of medical products relied on market surveillance and inspections.

Despite the successful pricing policies to reduce medicine prices, the state experienced significant losses due to fraud around 2007. Although falsification and theft contributed to these losses, all respondents indicated that the main reason for the implementation of the pharmaceutical track and trace system in Turkey (i.e., ITS) was the presence of “reimbursement fraud” or “barcode scamming.” Prior to ITS, pharmacies had to cut out the barcode of each product sold and put it behind the invoice. The invoice would then be sent to SGK for reimbursement. However, this system was vulnerable to fraud, as seen in the following example:

*I know your national identity number. I am a doctor and I am writing the prescription to you, but you don’t know that I am writing it. I give this prescription to the pharmacy. And the pharmacy doesn’t sell the drug but sells the barcode. It prints the barcode on the offset and sticks that barcode behind that prescription or the invoice and sends it to SGK. Takes the money, but there is no transaction or trade. I mean, nobody sells anything, but gets the money from SGK. It is not a fraud to people; it is a fraud to the government. —*Technical agency official

Respondents mentioned the existence of “printing houses” exclusively printing these falsified barcodes to be reimbursed by SGK. This fraud was estimated to account for US$1 billion annually.^31^^,^^32^

### Implementation of ITS

To prevent reimbursement fraud, the government decided to implement ITS. The first discussions on ITS occurred in 2007, but it took 3 years to convince and prepare stakeholders. The implementation took place in 2 phases to reduce implementation problems. A few months prior to the implementation of the first phase, a short pilot study was done with a small number of pharmacies. Then, phase 1, which focused on the manufacturers and pharmacists, was introduced in 2010. These 2 stakeholder groups, which are the front- and tail-end of the pharmaceutical supply chain, were obliged to make sales notifications, but wholesalers were not yet included in the system. Respondents mentioned that the phase 1 system, which corresponded to a point-of-dispense check system, remained vulnerable to introduction of falsified medicines at points in the supply chain where transactions were not tracked.

The initial implementation of phase 1 was problematic because of the way in which the software was developed. A single software engineer who had little experience in building enterprise systems was given the responsibility to create the software. The system crashed shortly after phase 1 was launched in 2010 and the system then had to be rebuilt from scratch by another person. Respondents indicated that the political will and determination of senior political figures were the main driving forces for this project to succeed:

*The undersecretary general called me to SGK, I was shopping in my sportswear at that time. He told me: “Okay, it is not important, come here!” I was running in the hall of SGK and I opened the door of the meeting room. I was sweating, in sportswear and I saw that two ministers, two undersecretary generals, two vice presidents, a lot of big guys were in the meeting room. I was shocked. The undersecretary general said: “This is the person I mentioned.” I sat between two ministers and they asked: “The system crashed, what should we do?” I told them: “First, accept it. In front of the news, in front of the media, first accept it and postpone it.” They said: “This is politics, we cannot do that. You have 10 days, please make it work.” This is the Eastern culture. —*Technical agency official

Respondents indicated that the political will and determination of senior political figures were the main driving forces for this project to succeed.

Phase 2 was implemented in 2012. It can be described as full track and trace, encompassing all actors within the domestic regulated supply chain. It was based on cross-checking movements of a product between each actor by comparing sales and purchase notifications. After phase 2, maintenance and development of ITS came under the responsibility of another company and MOH. The data on the medicines were pooled in a centralized database managed by the MOH. A schematic representation of the ITS workflow is shown in the Figure.

**FIGURE.**
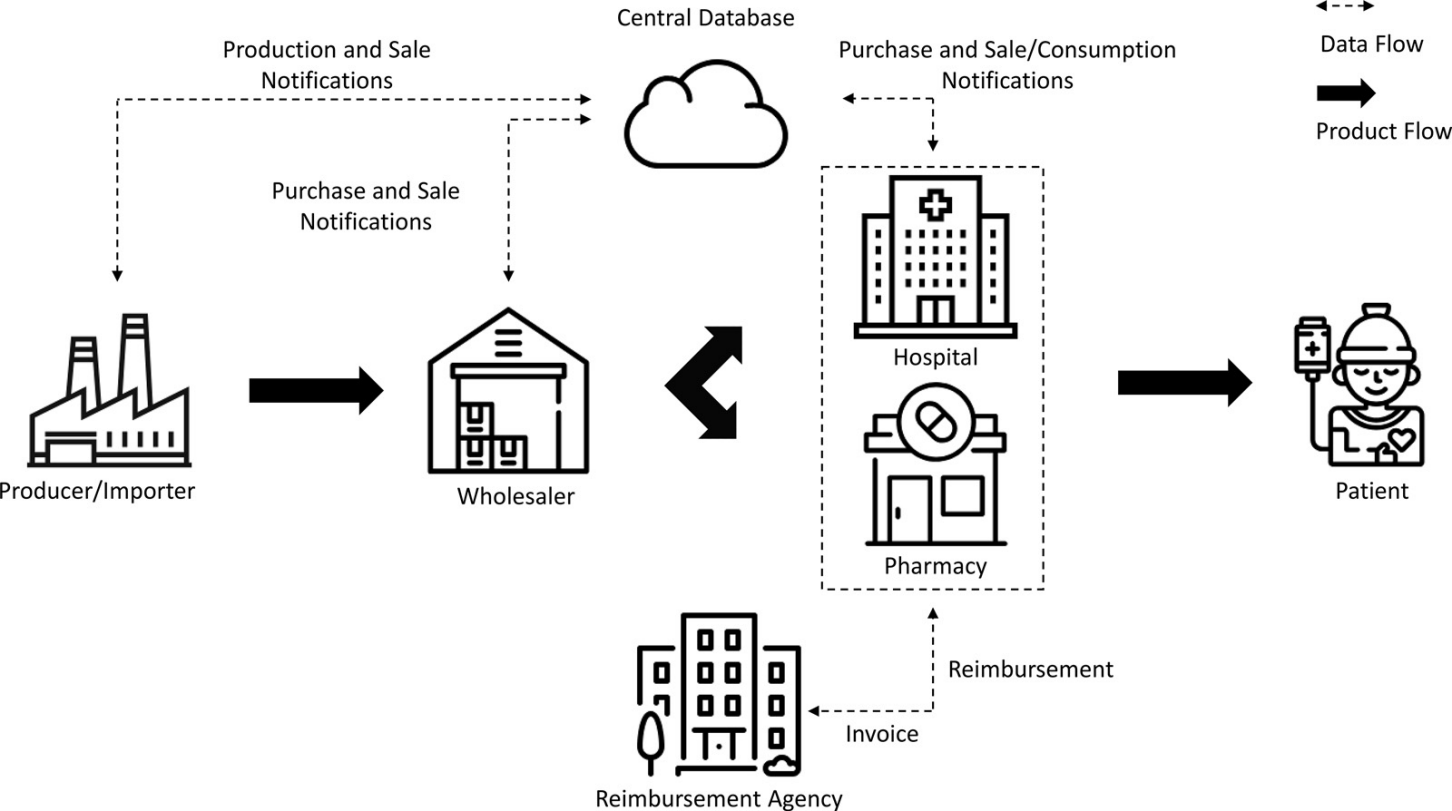
Simplified Schematic Representation of the Workflow of the Full Pharmaceutical Track and Trace System Used in Turkey

### Reaction to ITS

When ITS was first introduced, the expectations on its feasibility varied widely between different actors. Government institutions, including MOH and SGK, were convinced that the system would succeed, and that it would cut fraud and thus expenses in the national health system. Manu-facturers, however, did not think it was feasible. No other country had successfully executed national track and trace, and manufacturers were especially skeptical that it could be achieved in the very limited timeframe envisaged by Turkish politicians:

*There was a thing like: “Well, it won’t be implemented here anyways. It will fail. Let’s act as if we are complying to it, it won’t work anyways.” —*Multinational manufacturer

When ITS was introduced, the expectations on its feasibility varied widely; government institutions were convinced it would succeed, while manufacturers had doubts.

Industry was unhappy about having to bear the costs of compliance. One manufacturer estimated that his company invested around US$5 million in adding track and trace to their production and distribution flow, and that amount did not account for production losses. Another manufacturer said that the costs were around US$100,000 per conveyor belt. An additional concern of manufacturers was the lack of adequate equipment:

*We invited the Germans and Italians, who were good in machines. We talked to them. When we first bought it, we were buying dreams. (…) The ones who were able to convince you the most, you picked, because there was nobody that could show you [how it would work]. —*Multinational manufacturer

The wholesaler, who shared these initial doubts and skepticism, explained that they invested around 100 million Turkish Liras to implement this system across Turkey.

The merger of the 3 state payers into a single health insurer provided the state with consolidated buying power covering around 95% of the market, which was sufficient to incentivize the pharmaceutical industry to implement the system. Manufacturers also saw a benefit in reducing access to the market for medicine falsifiers, thus protecting income and value.

The pharmacists included in our study indicated that they were most concerned about the increased workload because each product had to be individually scanned. Forgetting to do so could have serious consequences for the pharmacists during inspections.

With all these different interests, the challenge of aligning personal and institutional incentives was far greater than any technological challenge, as an MOH official pointed out:

*I can get four people from India who can build this system in a short amount of time. The most crucial part is aligning all stakeholders with the support of the government. —*MOH official

The challenge of aligning personal and institutional incentives was far greater than any technological challenge.

### Implementation and Adaptation

Despite the phased implementation, stakeholders experienced many implementation problems, both physical and software related. In phase 1, issues with software development and realistic planning of the implementation process were experienced by manufacturers, wholesalers, and pharmacists. Additionally, a manufacturer explained that physically adapting the production lines to print and scan DataMatrix codes turned out to be challenging:

*We experienced a lot of problems. The ink got wiped because it did not dry properly. Also, when the conveyor belt was a bit skewed, the scanner could not read the DataMatrix [code]. —*Multinational manufacturer

Existing production lines in factories were not designed to be adapted, while new production lines were not yet developed. In addition, manufacturers experienced problems with sales notifications. Originally, the DataMatrix code on each secondary medicine package—the packaging enclosing the primary packages (e.g., blister or bottle)—had to be scanned individually, which took time and resources. Therefore, manufacturers introduced a “minimum-order-quantity system,” in which wholesalers were obliged to purchase medicines in fixed amounts. This system allowed manufacturers to scan the DataMatrix code on the tertiary packaging (i.e., the shipping-level packaging surrounding the secondary packaging).

Similarly, changes were made to logistic units.^25^ The industry realized that the serial shipping container code was more appropriate than the serialized global trade item number, which MOH initially proposed. However, respondents pointed out that MOH was willing to modify the system accordingly:

*The things that the authority sees from above and the reality we work in at the operational level are different. … We realized these things by experience. Therefore, things that were written down in theory evolved towards the reality of daily life in the end. Otherwise, if they did not change, if the ministry of health did not take our feedback into consideration, this system would be a nonoperative system. —*Multinational manufacturer

The wholesaler experienced similar implementation problems. Scanning the DataMatrix codes and adapting conveyor belts proved challenging. Also, staff needed to be trained to use the system appropriately. Most of the problems experienced by pharmacists related to software malfunction. When the system was inaccessible, pharmacists were not able to sell their products to patients. The problems were more acute in hospitals, where scanners were often in short supply. Additionally, hospitals buying common medicines in bulk found it difficult to scan individual prescriptions because the DataMatrix codes were not affixed to the packages of pills given to the patients but were only on the outer (or secondary) packaging, which was often thrown away before medicines were dispensed as individual patient prescriptions. As a result, hospitals were making consumption notifications instead of sales notifications.^26^

Although MOH was open and collaborative in adapting the system according to the needs of those involved during implementation, solving practical problems remained largely the responsibility of stakeholders.

### Outcomes of ITS

Respondents indicated that implementing ITS has had 5 main positive outcomes. First, reimbursement fraud is highly unlikely to happen in the current system. Successful fraud would require the participation of a long chain of people, raising the risk and reducing the reward for fraudsters. Reimbursement agency officials added that fraud cannot be reduced to zero but only minimized to a certain level, which is believed to have currently been achieved.

ITS has had several positive outcomes including reducing fraud, eliminating falsified medicines, optimizing recalls, empowering consumers, and matching supply to demand.

Second, ITS has largely eliminated falsified medicines in the regulated domestic supply chain. According to respondents, it is currently close to impossible to sell falsified medicines to patients in pharmacies and get reimbursement from SGK. Medicines cannot be “injected” into the supply chain at any stage other than by the manufacturer or importer. The only possibility for selling falsified products to patients is through out-of-pocket payments. However, since SGK provides comprehensive coverage to almost the entire population, citizens have no incentive to look outside the regulated supply chain or buy products out-of-pocket. Respondents underlined that Turkey’s health financing system, which reimburses pharmacists for verified dispensing and prescriptions, is central to the success of ITS. If pharmacists do not scan the DataMatrix code at dispensing, which serves to verify the authenticity of the product, SGK will not pay them for the product. In the case of a prescription medicine, SGK also verifies the authenticated product with the patient’s medical prescription before paying the pharmacist.

Third, respondents said that ITS has optimized the recall process for products that are degraded or show unwanted side effects. ITS enables quick and targeted recalls of specific products. The sale of suspect products can also be blocked within the system by MOH officials,^24^ which prevents further dissemination of poor-quality medicines and potentially significantly reduces public health harm caused by them.

Fourth, a mobile application of ITS, which was launched in 2014, allows citizens to scan the DataMatrix code of products. Citizens can immediately check the legitimacy of the product and obtain additional information, such as the expiry date, price, and recall status. Also, side effects can be entered in the application, which facilitates the collection of pharmacovigilance data.

Fifth, since ITS registers sales throughout the supply chain and eventual dispensing by outlet, the system allows for close monitoring of medicine stocks by health authorities, as well as providing inventory control for manufacturers, wholesalers, and pharmacies.

Although ITS has had many positive outcomes, respondents noted that ITS does not guarantee product quality. If a product has poor quality at manufacture or import, it will continue through the supply chain; careful tracking also does not protect against degradation. However, by reducing time spent on pharmacy inspections, ITS allows the transfer of human resources to other quality assurance functions:

*Before ITS, we had 3,000 inspectors [checking pharmacies]. After ITS, we have 100 inspectors. The other ones, we didn’t fire them, the other ones are used inside a new department which is GMP compliance, GDP [Good Distribution Practice] compliance and they are taking more samples from the market. They are going to the manufacturing sites and inspecting for substandard products. They are inspecting the active pharmaceutical ingredients. Now, they have time to inspect these things. —*Technical agency official

Although quantitative data are not available, respondents reported that the tracking capability of ITS in combination with sufficient and qualified human resources has significantly increased the possibility of detecting substandard and falsified medicine.

### Future Adaptations to ITS

Respondents suggested 2 potential improvements to ITS. First, the scope of products given a DataMatrix code should increase. Currently, almost all medicines under the responsibility of MOH are obliged to carry a DataMatrix code, and internet sales are prohibited. However, some products, such as intravenous and radiopharmaceutical products, active pharmaceutical ingredients, and personalized medicines compounded in the pharmacy, are excluded from the DataMatrix code requirement. In addition, products such as vitamins and dietary supplements that are overseen by the Ministry of Food, Agriculture, and Livestock are not included in ITS. Respondents pointed out that falsification currently takes place with over-the-counter products rather than prescription medicine because inspections and regulations are less rigid. Although the majority of patients are aware that quality cannot be guaranteed, some products such as weight loss products, sexual products, or dietary supplements are sometimes purchased on the internet.

ITS could be improved by expanding the scope of monitored products and by developing a proactive alarm system to prevent stock-outs and shortages.

Second, an MOH official mentioned that ITS data could be used more effectively to prevent shortages and stock-outs. Although the current system is largely reactive, MOH aims to implement a proactive alarm system that provides a warning when the supply of a certain product goes below a specific threshold in a particular area. Such warnings will enable the system to procure medicine more rapidly and to prevent drug shortages more successfully.

## DISCUSSION

Several countries and regions have attempted to introduce full pharmaceutical track and trace systems. Turkey was the first to succeed. This study aimed to elucidate the factors underpinning the success of this technological innovation. We find that the drivers of success were more political and economic than technological.

China, which has considerable experience and capability in implementing large technological programs in its health sector, suspended plans to introduce pharmaceutical track and trace in 2016 after facing considerable resistance from medicine manufacturers. Industry was concerned that the linear barcode system proposed instead of a DataMatrix code would create a large footprint on the medicine package to capture the required data. Further, there was concern that the requirement that all barcodes be printed only by the government would result in a burdensome and costly procedure for manufacturers.^33^ As discussions with stakeholders continue, the target data for implementation has been pushed back to 2022.

The United States provides another example in which difficulty in adequately aligning incentives for all key actors has led to slow adoption of full traceability. Industry has not fully complied with the phased implementation foreseen in the 2013 Drug Supply Chain Security Act. Implementation of the act is expected to take a full decade.^4^^,^^34^

How was Turkey, a middle-income country with no great tradition of technological innovation, able to succeed where others stumbled? The most critical element was the combination of a widely recognized problem and political determination to solve it.

How was Turkey, a middle-income country with no great tradition of technological innovation, able to succeed where others stumbled?

The winners of the 2002 elections in Turkey sought to establish political legitimacy through programs that delivered benefits to a broad swath of citizens. One of these benefits was universal health coverage delivered through a single-payer state institution. When high levels of fraud threatened the sustainability of this coverage, politicians threw their weight behind an ambitious technological solution within an improbably tight timeframe.

The state controlled access to a large and expanding pharmaceutical market, and manufacturers who wanted to sell into that market had to play ball. A generous benefit package greatly reduced out-of-pocket spending on medicines. Together with a prohibition on internet sales of prescription products, the benefit package removed any incentive for patients to purchase products from the unregulated supply chain. At the same time, the reimbursement system obliged pharmacists to bow to the will of the government; if they did not, they would not get paid. Together, these factors allowed for the widespread adoption of the system.

The successful implementation of the system was underpinned by another key factor: a willingness of the government, which was driving the process, to support flexible and adaptive solutions to problems identified during implementation. These work-arounds were not just technical; like all adaptive implementation, they also had a social component, encompassing human actions and relations.^18^ The Turkish state mainly focused on facilitating the social component, while other actors took responsibility for implementing the technical components of ITS.

Turkey’s centralized database allowed for verifying reimbursement data because its track and trace database was linked to the database of SGK, the single-payer state-owned reimbursement agency. This process enabled reducing fraud dramatically.^8^ Centralized databases rely heavily on the presence of sufficient technical capacity at the central level. If this capacity is lacking, outsourcing the development of the system to a software company, as in Turkey, might solve the problem, as long as security and privacy concerns of stakeholders are addressed.^4^ In environments without political power emerging from a single-payer institution, the reimbursement landscape might be fragmented. The reimbursement agencies within a fragmented market, as well as the pharmaceutical industry, might oppose sharing and centralizing their competitive data more strongly.^35^^,^^36^ In these circumstances, setting up a distributed database that gives stakeholders more authority over their data might be more feasible. However, disadvantages of distributed databases include difficulty in governing and adapting the system because ownership of the data is not centralized.^4^^,^^8^

To our knowledge, this study is the first to evaluate the emergence, implementation, and outcomes of ITS in Turkey, while focusing on the underlying political and economic factors. Since Turkey is the first country in the world to implement a full track and trace system, the implications of this study might be of particular interest to countries aiming to implement similar track and trace systems, including the EU member states, China, and the United States.

The implications of this study might be particularly interesting for countries aiming to implement track and trace systems, including the EU member states, China, and the United States.

### Limitations

The findings of this qualitative study could be strengthened through triangulation with quantitative data on the quality of medicine in the Turkish pharmaceutical market, the implementation costs of ITS, and the effect of ITS on public health. However, attempts to verify estimates provided by respondents with quantitative data from government or other formal sources proved unsuccessful. Future studies on the cost effectiveness of ITS might provide valuable insights.

For some categories of participants, we interviewed only a single key informant. However, triangulation of data provided by respondents from different sectors (e.g., manufacturers, whole-salers, technical agencies) in combination with further triangulation from literature increases our confidence in the validity and reliability of our study data.

## RECOMMENDATIONS

The outcomes of our study show 3 main implications for countries aiming to implement pharmaceutical track and trace systems.

First, a track and trace system should be seen as a means to an end, rather than a goal in itself. To function, it must be underpinned by well-defined legislation and regulatory capacity, including laboratories and frequent GMP and GDP inspections. Without these, there is a risk of “garbage in equals garbage out.” In that case, track and trace may simply deliver a secure supply chain for poor-quality products.

Second, the incentives of all the stakeholders need to be aligned to successfully adopt the system. The role of the state should not be underestimated. It should both facilitate the implementation process with its political power, as well as provide sufficient leeway to adapt the system according to the needs of stakeholders. Countries lacking powerful political leadership might expect greater resistance to implementing pharmaceutical track and trace systems from stakeholders. This resistance is especially likely from stakeholders that bear the burden of upfront investment in technology and those that might benefit from gaps in the supply chain.

Third, countries/regions should aim to implement a full track and trace system. Although the benefits of track and trace systems are not universal and rely on the nature of the pharmaceutical system of the implementing country, point-of-dispense check systems, which exclude wholesalers and other middlemen, preclude some of the more important benefits of full track and trace. They do not provide data to flag regional shortages. Further, because they do not allow for traceability of products throughout the supply chain, such partial systems limit the ability to recall products. As a result, falsified products might circulate in a market for months without detection.^3^ This situation is especially true in the EU’s complex single-market supply chains. Although the European Medicines Verification System has added an antitampering device to the outer medicine package to prevent unlawful repackaging, nonreimbursed or over-the-counter products will remain vulnerable to falsification. Most importantly, in EU member states lacking closed supply chains, patients might buy products online that are less likely to be verified and may even be excluded from verification. Although accreditations, domain name verifications, and logos for online pharmacies exist, their effectiveness can still be undermined by a lack of consumer awareness, vulnerability to misuse, unavailability of certain types of products at accredited online pharmacies, and the attractiveness of cheaper options.^37^^–^^39^

## CONCLUSION

Although track and trace systems are sometimes presented as reproduceable technical solutions to quality assurance in the supply chain, this study shows that the main drivers of success for ITS in Turkey were highly dependent on the presence of a specific set of circumstances. These included political determination induced by reimbursement fraud, political power emerging from a single-payer institution that generated a substantial pharmaceutical market, reimbursement for verified dispensing and prescription, and flexibility to adapt the system according to the needs of stakeholders during implementation.

Despite ITS’s success in providing a clean regulated supply chain, it represents only part of the solution. ITS can only operate effectively if it is embedded in a pharmaceutical market where all legislative and regulatory components are in place.

## Supplementary Material

20-00084-Parmaksiz-Supplement_2.pdf

20-00084-Parmaksiz-Supplement_1.pdf

## Notes

**Peer Reviewed**

**First published online:** August 19, 2020
