# Corrigendum: Parmaksiz K et al., What Makes a National Pharmaceutical Track and Trace Succeed? Lessons From Turkey

**DOI:** 10.9745/GHSP-D-20-00587

**Published:** 2020-12-23

See corrected article.

In the article “What Makes a National Pharmaceutical Track and Trace Succeed? Lessons From Turkey” by Koray Pamaksiz et al. (Volume 8, Issue 3), the Funding statement on page 10, incorrectly listed the U.S. Pharmacopeia Quality Institute as one of the funders of the work.

The article has been corrected accordingly.

## Notes

© Global Health: Science and Practice. This is an open-access article distributed under the terms of the Creative Commons Attribution License, which permits unrestricted use, distribution, and reproduction in any medium, provided the original author and source are properly cited. To view a copy of the license, visit http://creativecommons.org/licenses/by/4.0/. When linking to this article, please use the following permanent link: https://doi.org/10.9745/GHSP-D-20-00587

