# Supplementary material for: What Makes a National Pharmaceutical Track and Trace System Succeed? Lessons From Turkey
Source: Glob Health Sci Pract. 2020 Sep 30;8(3):431–41. doi: 10.9745/GHSP-D-20-00084 (PMC7541108; doi:10.9745/GHSP-D-20-00084)
Supplement: 20-00084-Parmaksiz-Supplement_2.pdf [file 20-00084-Parmaksiz-Supplement_2.pdf]

Supplement to: Parmaksiz K, Pisani E, Kok MO. What makes a national pharmaceutical track and trace system succeed? Lessons from Turkey. *Glob Health Sci Pract*. 2020;8(3). https://doi.org/10.9745/GHSP-D-20-00084

Coding Tree

Primary Code	Secondary Code	Tertiary Code	Quaternary Code	Quinary Code
00_Political_economy				
	Economic_factors	Jobs		
		National_income		
	Political_factors	Lobbying		
		Political_promises		
		Political_will		
01_Market_opportunity				
	1_Supply_side	Conflict_or_disaster		
		Distorting_regulation		
		Infrastructure		
		Limited_manufacturing_capacity		
		Procurement_practices		
		Producer_incentives		
	2_Demand_side	Affordability_out_of_pocket		
		Affordability_UHC_insurance		
		Incentive_structures		
		Patient_preference		
		Unexpected_demand		
	3_Market_regulation	Price_related		
			Global_budget	
			Reference_pricing	Future adaptations

			Consequences
			Functioning
			Reaction_towards_RP
		Trade_related	
02_Market_dynamics			
	Export		
	Import		
	Parallel_trade		
	Transit		
03_Actors			
	1_Macro	Government	
		MOH	
		Organised_crime	
		Other_international_org	
		WHO	
	2_Producers (see also 9)		
	3_Supply_chain	Brokers	
		Distributors	
		Smugglers	
		Wholesalers	
	4_Healthcare_professionals	Hospital	
		Insurers	
		Pharmacy	
	5_Patient_market_interface	Internet	
		Market	

	6_Regulators	MRA
		Police
04_Facilitator		
	Corruption	Protecting_economic_interests
	Disruption_&_adaptation	New_technologies
		Pricing_and_or_medicine shortage
	Limited_capacity	Financial_resources
		Human_resources
		Technology
	Systemic_failure	No_due_dilligence
		Poor_coordination
		Poor_planning
05_Motivator		
	1_Legal_profit	Arbitrage
		Competitive_advantage
		Cost_reduction
	2_Illegal_or_liminal_profit	Access_to_meds
		Avoid_red_tape_or_laziness
		Fraud_&_money_laundering
		Need
		Perverse_incentives
06_Deterent		
	1_Product_regulation	GDP
		GMP
		Inspection

		Licensing_&_market_authorisation		
		Standard operating procedures		
	2_Detection	Laboratory		
		Reporting_systems		
		Risk_assesment		
		Track_and_trace	Future adaptations	
			Consequences	
			Costs	
			Functioning	
			Implementation_problems	Physical_problems
				Software_problems
			Reactions_towards_track&trace	
	3_Transparency			
	4_Systemic_approach	Avoid_production_shortages		
07_Legislation				
	Definitions			
	Medicine-related-laws			
	Penalties	Enforcement		
		Penalties_for_other_falsification		
		Penalties_for_pharma_crime		
08_Quality				
	Falsified	Criminal_substandard		
		Expiry_date_extended		
		Quality_but_falsely_labelled		
		Repackaged_as_different_product		

		Total_fake
	Other_illegal	Stolen_or_diverted
		Unregistered
	Substandard	Degraded
09_Category_of_meds		
	Producer_type	API
		Generic
		Innovator
	Therapeutic	Lifestyle
		Noncommunicable diseases
		Other
		Sexual_health
		Vaccines

Abbreviations: API, Active Pharmaceutical Ingredient; GDP, Good Distribution Practice; GMP, Good Manufacturing Practices; MOH, Ministry of Health; MRA, Medicines Regulatory Authority; RP, Reference Pricing; WHO, World Health Organization.
