# Supplementary material for: What Makes a National Pharmaceutical Track and Trace System Succeed? Lessons From Turkey
Source: Glob Health Sci Pract. 2020 Sep 30;8(3):431–41. doi: 10.9745/GHSP-D-20-00084 (PMC7541108; doi:10.9745/GHSP-D-20-00084)
Supplement: 20-00084-Parmaksiz-Supplement_1.pdf [file 20-00084-Parmaksiz-Supplement_1.pdf]

Supplement to: Parmaksiz K, Pisani E, Kok MO. What makes a national pharmaceutical track and trace system succeed? Lessons from Turkey. *Glob Health Sci Pract*. 2020;8(3). https://doi.org/10.9745/GHSP-D-20-00084

Interview Guide

The interviews were adjusted according to the interviewee and their area of expertise.

The interviews started off with background information about the interviewer and the purpose of the research. These were followed by introductory questions about the interviewee.

Theme 1: Pharmaceutical Track and Trace in Turkey

1. Could you elaborate on the history of the pharmaceutical market before pharmaceutical track and trace (ITS) was implemented?
    a. How were pharmaceuticals regulated?
    b. Was there a problem of substandard and falsified medicines?
    c. What were the main drivers of the government for implementing ITS? (e.g., scandal, costs, inefficiencies)
2. How was ITS implemented?
    a. In what stages was ITS implemented?
    b. What were the problems stakeholders faced during this implementation process?
3. Differences between implementation and practice of ITS
    a. Which adaptations were made to the original plan of implementation after challenges were faced during implementation?
        i. What do you think the consequences of these adaptations are?
    b. Do you have an estimation of actual the costs of the implementation?
    c. Which areas does ITS cover? (e.g., Active Pharmaceutical Ingredients, imported medicine, pharmacy compounded pharmaceuticals, internet purchases)
    d. What are the areas ITS should improve on?
        i. Could you give examples?
4. Consequences of ITS on substandard and falsified medicines
    a. How did ITS affect the production, distribution, and sale of substandard and falsified medicines?
    b. What are the costs and benefits of ITS and in which areas are they mainly realized? (e.g., reducing human errors, optimizing supply chain)
    c. There are still reports about the police force regularly confiscating substandard and falsified medicines in Turkey. How would you explain this?
        i. What are the effects of ITS on the unregulated supply chain? (e.g., black market, internet)
        ii. Do you think ITS has shifted the problem of substandard and falsified medicines from the domestic market to the international market, or are there still substandard and falsified medicines produced for the domestic market?
        iii. Are there other methods, next to ITS, that Turkey implemented or plans to implement to combat substandard and falsified medicines?
5. How do you think pharmaceutical regulation (e.g., long approval times, restricting laws) affects the entry of substandard and falsified medicines?

Theme 2: The Pharmaceutical Market in Turkey

1. <u>Demand</u>
    a. How do you think patient preferences (originator vs generic) influence the entry of falsified and substandard medicines into the supply chain in Turkey?
        i. Are falsified medicines often originator or generic products?
    b. How do you think the current incentive structure for physicians prescribing pharmaceuticals affects the demand for pharmaceuticals?
        i. Do you think this creates an opportunity for substandard and falsified medicines to enter the supply chain? And if so, how?
    c. Does the current universal health coverage cover all pharmaceuticals?
        i. On what basis does Turkey decide which medications to cover? (e.g., price, demand)

2. <u>Supply</u>
    a. Financial factors
        i. Was there a downward pressure on pharmaceuticals after scaling up of health coverage?
            1. If yes, how did pharmaceutical manufacturers react?
            2. If yes, did this pressure on price affect the quality of medicine?
        ii. If no, how did Turkey manage to contain costs?
        iii. Do you think reference pricing is a suitable tool for controlling drug expenditures in Turkey?
            1. What are the downsides of the reference pricing system? (e.g., exchange rates, selected countries)
            2. What is the reaction of pharmaceutical manufacturers toward the reference pricing system?
            3. What alternatives, other than external reference pricing, could/should be used to control drug expenditures?
    b. Producer (dis)incentives to enter market
        i. What are the main barriers for producers to enter the pharmaceutical market in Turkey? (e.g. price, regulation, laws)
        ii. *I read there is a tendency towards "domestic production" of pharmaceuticals in Turkey recently*. What incentives is Turkey planning to give producers to enter the market?
    c. Manufacturing capacity
        i. Do you think Turkey's current manufacturing capacity is sufficient to supply the domestic market?
        ii. If not, how does Turkey guarantee the quality of its import?
            1. Does it require additional documentation from the exporting country? (e.g. standards, certificates, GMP)
            2. How does Turkey guarantee quality control of Active Pharmaceutical Ingredients?

3. Dynamics
    a. Import and export
        i. *Currently, Turkey is importing more pharmaceuticals than it is exporting*. Do you think this will cause challenges for pharmaceutical supply in future?
        ii. What do you think the effect of this trade gap will be on substandard and falsified medicines?
        iii. Does the Turkish regulator regulate products that are exported?
            1. If not, what do importing countries demand in the way of quality control/GMP certification?
    b. Transit
        i. How do you think Turkey's geographical location (as a country bridging Europe and Asia) affects the transit of substandard and falsified medicines?
        ii. What are particular measures Turkey takes to combat this?

Conclusion

Do you have any questions regarding this interview?

Are there any points you just made that you would like to comment on or emphasize?

Thank you for your time and valuable insights.
